# NF-Y Subunits Overexpression in HNSCC

**DOI:** 10.3390/cancers13123019

**Published:** 2021-06-16

**Authors:** Eugenia Bezzecchi, Andrea Bernardini, Mirko Ronzio, Claudia Miccolo, Susanna Chiocca, Diletta Dolfini, Roberto Mantovani

**Affiliations:** 1 Dipartimento di Bioscienze, Università degli Studi di Milano, Via Celoria 26, 20133 Milano, Italy; eugenia.bezzecchi@unimi.it (E.B.); andrea.bernardini@unimi.it (A.B.); mirko.ronzio@unimi.it (M.R.); diletta.dolfini@unimi.it (D.D.); 2 Department of Experimental Oncology, IEO, European Institute of Oncology IRCCS, Via Adamello 16, 20139 Milan, Italy; claudia.miccolo@ieo.it (C.M.); susanna.chiocca@ieo.it (S.C.)

**Keywords:** HNSCC, NF-Y, transcription factors, TCGA, CCAAT box, alternative splicing

## Abstract

**Simple Summary:**

Cancer cells have altered gene expression profiles. This is ultimately elicited by altered structure, expression or binding of transcription factors to regulatory regions of genomes. The CCAAT-binding trimer is a pioneer transcription factor involved in the activation of “cancer” genes. We and others have shown that the regulatory NF-YA subunit is overexpressed in epithelial cancers. Here, we examined large datasets of bulk gene expression profiles, as well as single-cell data, in head and neck squamous cell carcinomas by bioinformatic methods. We partitioned tumors according to molecular subtypes, mutations and positivity for HPV. We came to the conclusion that high levels of the histone-like subunits and the “short” NF-YAs isoform are protective in HPV-positive tumors. On the other hand, high levels of the “long” NF-YAl were found in the recently identified aggressive and metastasis-prone cell population undergoing partial epithelial to mesenchymal transition, p-EMT.

**Abstract:**

NF-Y is the CCAAT-binding trimer formed by the histone fold domain (HFD), NF-YB/NF-YC and NF-YA. The CCAAT box is generally prevalent in promoters of “cancer” genes. We reported the overexpression of NF-YA in BRCA, LUAD and LUSC, and of all subunits in HCC. Altered splicing of NF-YA was found in breast and lung cancer. We analyzed RNA-seq datasets of TCGA and cell lines of head and neck squamous cell carcinomas (HNSCC). We partitioned all TCGA data into four subtypes, deconvoluted single-cell RNA-seq of tumors and derived survival curves. The CCAAT box was enriched in the promoters of overexpressed genes. The “short” NF-YAs was overexpressed in all subtypes and the “long” NF-YAl in Mesenchymal. The HFD subunits are overexpressed, except Basal (NF-YB) and Atypical (NF-YC); NF-YAl is increased in p53 mutated tumors. In HPV-positive tumors, high levels of NF-YAs, p16 and ΔNp63 correlate with better prognosis. Deconvolution of single cell RNA-seq (scRNA-seq) found a correlation of NF-YAl with Cancer Associated Fibroblasts (CAFs) and p-EMT cells, a population endowed with metastatic potential. We conclude that overexpression of HFD subunits and NF-YAs is protective in HPV-positive tumors; expression of NF-YAl is largely confined to mutp53 tumors and malignant p-EMT cells.

## 1. Introduction

Squamous cell carcinomas of the head and neck (HNSCC) are major medical concerns worldwide [1], associated with high mortality and morbidity, as the survival outcome of these patients has remained poor over the past decades. They are a heterogenous group of tumors arising from the transformation of keratinocytes of the oral cavity and the upper respiratory tract, including the nasopharynx, paranasal sinuses, oropharynx, hypopharynx and larynx. The main risk factors are environmental (tobacco and alcohol) and human papillomavirus (HPV) infections. According to several parameters (gene expression, pathways profiling and clinical features) HNSCC is classified into four subtypes: Basal, Mesenchymal, Atypical and Classical [2,3,4,5,6].

In general, cellular transformation is a consequence of genetic mutations, entailing changes in the patterns of gene expression. This is ultimately obtained through alterations in the structure, expression levels or usage of sequence-specific transcription factors (TFs) and chromatin-modifying cofactors. Indeed, it is known that changes in the structure or expression levels of oncogenic TFs predispose, or directly cause, profound changes in gene expression, leading to tumorigenesis. Sequence-specific TFs bind to short elements within the promoters and enhancers of genes [7]. Two-decade-long efforts devoted to the discovery of genes overexpressed in different types of cancer have led to the identification of transcription factor binding sites (TFBSs) in their promoters. This was achieved by analyzing the expression profiling, first by microarrays and, more recently, by RNA-seq. In numerous reports, the CCAAT box was identified as enriched in promoters of “tumor” genes, including in a widespread analysis of >60,000 tumor samples [8]. This element is located in promoters at −60/100 from the transcription start sites, and a wealth of genetic studies determined that it is important or crucial for the high-level expression of genes in vitro and in vivo [9]. NF-Y is a heterotrimer formed by the histone fold domain (HFD) dimer NF-YB/NF-YC, and the sequence-specific NF-YA, providing CCAAT specificity. NF-YA is present in two major isoforms, “short” and “long”, differing in 28 amino acids generated by alternative splicing of Exon 3 [10]. NF-YC is also present in multiple isoforms, resulting from alternative splicing at the *C*-terminus of the protein [11]. In both NF-YA and NF-YC, the heterogeneity involves Gln-rich *trans*-activation domains, whereas the subunits interaction and DNA-binding parts are identical in all isoforms. The NF-Y regulome has been studied and at least partially understood through chromatin immunoprecipitation (ChIP-Seq) and functional analysis (profiling/RNA-seq after inactivation) in different cell types. This exercise identified two common cancer-promoting pathways controlled by NF-Y: cell-cycle and metabolic genes [12,13,14,15,16,17], reviewed in [18].

NF-Y has long been considered a ubiquitous transcription factor present at similar levels in normal and transformed cells; this view has largely changed, in part because of recent studies on the mRNA expression of its subunits in cancer. Data in ovarian [19,20], breast [21] and gastric [22,23] cancers indicated overexpression of NF-YA in tumors. Examination of the levels of NF-YA in cancer specimens present in The Cancer Genome Atlas (TCGA) by Firebrowse (http://firebrowse.org/viewGene.html, accessed on 20 January 2020) suggested overexpression in epithelial tumors [24]. We thus started a systematic investigation of large RNA-seq datasets in cancers of epithelial origin, with analysis in the breast [24], lung [25,26] and liver [27]. Here, we focused on HNSCC. We completed the TCGA classification by including all 522 available tumors in the four molecular subtypes, we analyzed 35 HNSCC cell lines partitioned according to mutations and HPV status, we exploited single-cell RNA-seq data of primary tumors [28] to predict cell population composition of TCGA samples and we analyzed NF-Y expression. Several notable—and unexpected—features emerged.

## 2. Materials and Methods

### 2.1. TCGA and CCLE RNA-Seq Dataset

As of April 2021, RNA-seq data on 522 HNSCC primary tumors [6] and 44 non-tumor tissues were present in TCGA. We downloaded the non-normalized raw count data from http://firebrowse.org/ (accessed on 20 January 2020).

To extend the currently classified TCGA tumors in the 4 molecular subtypes, we used 838 genes previously validated as signatures for the 4 individual subtypes [3]; each gene was median-centered on all 522 HNSCC samples, and Pearson correlations were calculated between the predictor centroids and the TCGA samples. A subtype prediction for each tumor was given by the centroid based on the largest correlation value.

Analysis of the RNA-seq cell lines database was performed by retrieving all available paired-end FASTQ files of the upper aerodigestive tract cell lines from the Cancer Cell Lines Encyclopedia (SRA Study SRP186687), UM-SCC-4, UM-SCC-6, UM-SCC-19, UMSCC-47 (SRA Study SRP170971) and SCC-0472, SCC-090 (SRA Study SRP136016) from the Gene Expression Omnibus (GEO) repository. The data were processed as described above.

### 2.2. Global Gene Expression and Downstream Analyses

Cell lines RNA-seq data were analyzed with RSEM-1.3.1. to figure out expression of mRNA at genes and isoforms levels.

Differential gene expression analysis of RNA-seq data was performed using R package DESeq2 [29]. The tumor versus normal expression fold change (FC) denotes upregulation or downregulation according to the FC value. Log2FC and the corresponding false discovery rate (FDR) were reported by the R package. FDR < 0.01 and |log_2_FC| > 2 were set as inclusion criteria for selection as a differentially expressed gene (DEG) in tumors/subtypes versus normal samples. 

We used KOBAS 3.0 (http://kobas.cbi.pku.edu.cn/anno_iden.php, accessed on 20 January 2020) for pathway enrichment analysis using the ENTREZ gene IDs. *p* values of the enriched pathways were obtained by performing FDR correction. The TFBS analysis and de novo motifs were performed using Pscan software and Bonferroni corrected *p* values, as reported in [30].

### 2.3. Correlation between Mutational and HPV Status and NF-Y Levels

We downloaded data on somatic mutations (SNP and INDEL) from the MC3 public version on https://tcga.xenahubs.net (accessed on 20 January 2020) and we compared NF-YA, NF-YB, NF-YC, NF-YAl and NF-YAs levels in samples with 1 or more mutations in 1 gene with the rest of samples not presenting mutations in that gene. We retrieved the HPV status in the clinical table downloaded from the firebrowse.org website. *p* values were calculated with the Wilcoxon rank sum test.

### 2.4. Western Blot Analysis

As previously described [31], the UM-SCC-4, UM-SCC-6, UM-SCC-18, UM-SCC-19, UM-SCC-23 and UM-SCC-47 cell lines were obtained from Prof. Thomas E. Carey [32]. The UD-SCC-2 cell line was kindly provided by Prof. Henning Bier (present address: LRZ, Munich, Germany) [33]. The UM-SCC-104 cell line [34] was acquired from Merck; the UPCI:SCC-152 and UPCI:SCC-154 cell lines were from ATCC. Cells were grown in Dulbecco’s modified Eagle’s medium supplemented with antibiotics, 2 mM l-glutamine, 10% fetal bovine serum and non-essential amino acids. All cell lines were authenticated by short tandem repeat profiling and tested for mycoplasma contamination every 6 months.

Total protein extracts were prepared in RIPA buffer (150 mM NaCl, 1% NP-40, 0.5% DOC, 0.1% SDS, 50 mM Tris-HCl pH 8 and proteases inhibitors) and equal amounts of total proteins were resolved by SDS-polyacrilamide gel electrophoresis under reducing conditions. The membrane was probed with primary antibodies and the appropriate horseradish peroxidase conjugated secondary antibodies (Sigma Aldrich, St. Louis, MO, USA). The primary antibodies used were the anti-NF-YA (G2, Santa Cruz Biotechnologies, TX, USA), anti-NF-YB (GeneSpin, Milan, Italy) and anti-Vinculin (Sigma Aldrich, St. Louis, MO, USA) as loading controls. The signal was acquired with Biorad ChemiDoc. The uncropped western blotting figure can be found in Figure S5.

### 2.5. Analysis of Clinical Data

We retrieved clinical data related to the TCGA HNSCC samples, including progression-free interval (PFI) time records of 521 patients, from the https://xenabrowser.net/ (accessed on 20 January 2020). Survival analysis of samples stratified according to NF-Y (subunit/isoforms) levels was carried out with the survminer package in the R environment, according to Kaplan–Meier analysis and log-rank test [35,36]. The two groups analyzed refer to: low < first 3 quartiles; high > last quartile. For 2-genes combination survival analysis, we independently ranked samples according to the increasing expression of genes and split them into 2 groups: the Low label corresponds to the 75% of samples with lower expression; High to the remaining 25%. We obtained the combinations by joining the 2 rankings.

### 2.6. Deconvolution Analysis of TCGA Samples and Signature Correlation

Cell-type deconvolution on TCGA bulk RNA-seq samples was computed using the SCDC R package, using single-cell data published by Puram et al. [28] as reference datasets. For gene signature evaluation, the lists were retrieved from Puram et al. [28] and the Z-scores of the Log_2_ transformed TPM (Transcripts Per Million) of genes were computed. For the CAF1/2 signatures, Z-scores were calculated on Mesenchymal samples. For the p-EMT signature samples, samples with a predicted proportion of fibroblasts lower than 30% were selected. For each signature, the median Z-score was computed and assigned to each sample. According to the median Z-score, samples were ranked and split into High (top 40% of samples) and Low (lower 40% of samples); the 20% of intermediate samples were discarded. 

### 2.7. Statistical Analysis

Single comparisons between 2 groups (normal versus tumor/subtype-specific specimens and mutated versus wild-type samples) were performed with the Wilcoxon rank sum test, using the stat compare means function of the ggpubr package. In order to verify significant trends between EMT marker levels and NF-YAl/NF-YAs ratios, we divided all tumor samples into 10 groups with increasing ratio values, and then calculated the Jonckheere’s Trend Test using the JT. test function provided by the SAGx package. The charts were created in the RStudio environment (R version 3.6.3) with the ggplot2 and heatmap.plus packages.

## 3. Results

### 3.1. HNSCC Differentially Expressed Genes (DEGs) Have CCAAT in Their Promoters

**Figure 1:**
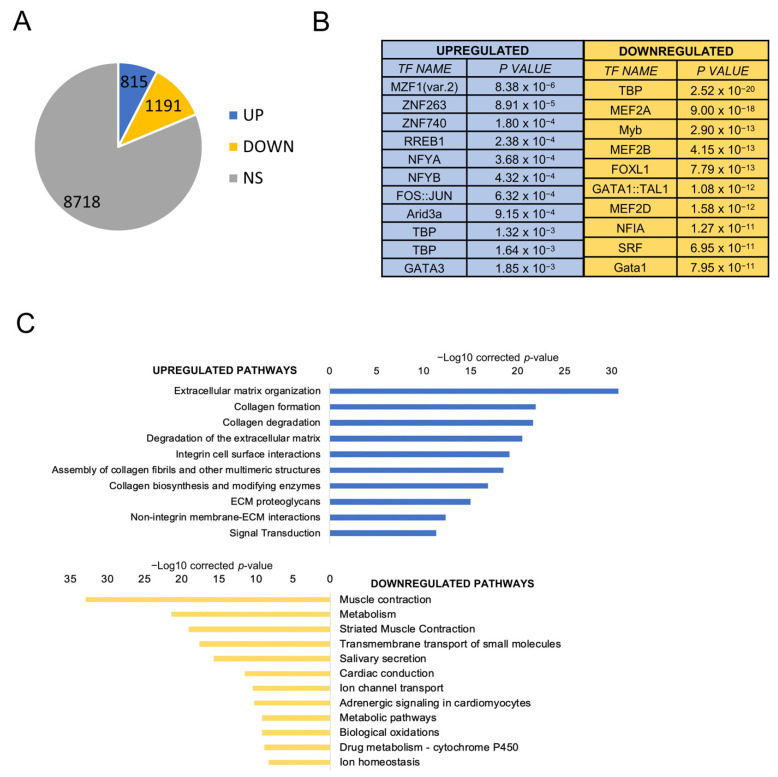
Gene expression analysis of HNSCC TCGA tumors. (**A**). Up- and downregulated genes in HNSCC versus normal tissues. NS, not significant. (**B**). Pscan analysis of enriched TFBS in promoters (−450/+50 bps from the TSS) of up- and downregulated genes in HNSCC. (**C**). Reactome pathways enriched in upregulated genes (upper panel) and downregulated genes (lower panel) listed according to their *p* value. The list was obtained using KOBAS.

Nothing is known about the prevalence of CCAAT boxes in the promoters of HNSCC differentially expressed genes. We downloaded all 522 RNA-seq datasets present in TCGA [6] and compared the gene expression levels of tumors to those of the available normal samples. Using a |log_2_FC| > 2, FDR < 0.01 threshold, 815 genes were found to be overexpressed and 1191 to be downregulated (Figure 1A), listed individually in Table S1. To gather information on regulatory DNA elements, we analyzed promoters (−450 to +50 from the transcriptional start site) with the Pscan software [30]. At the top of the list of overexpressed genes, we found logos of Zn Finger Transcription Factors (MZF, ZNF263, ZNF740, RREB1), with the NF-Y matrix (NFYA/NFYB) coming immediately afterward (Figure 1B, left panels). As for downregulated genes, other matrices (TBP, MEF2, FOX and NFI) were found, with robust *p* values (Figure 1B, right panels). We then used the KOBAS software to identify Gene Ontology (GO) terms in DEGs: in upregulated genes, extracellular matrix terms (collagen formation/degradation, integrins) predominate (Figure 1C); signal transduction was enriched, but cell-cycle terms were absent, scoring a clear difference from other epithelial tumors we analyzed previously. The presence of metabolism terms among the downregulated genes is also remarkable, along with muscle, which is not immediately obvious to explain in this context.

### 3.2. NF-Y Overexpression in HNSCC

**Figure 2:**
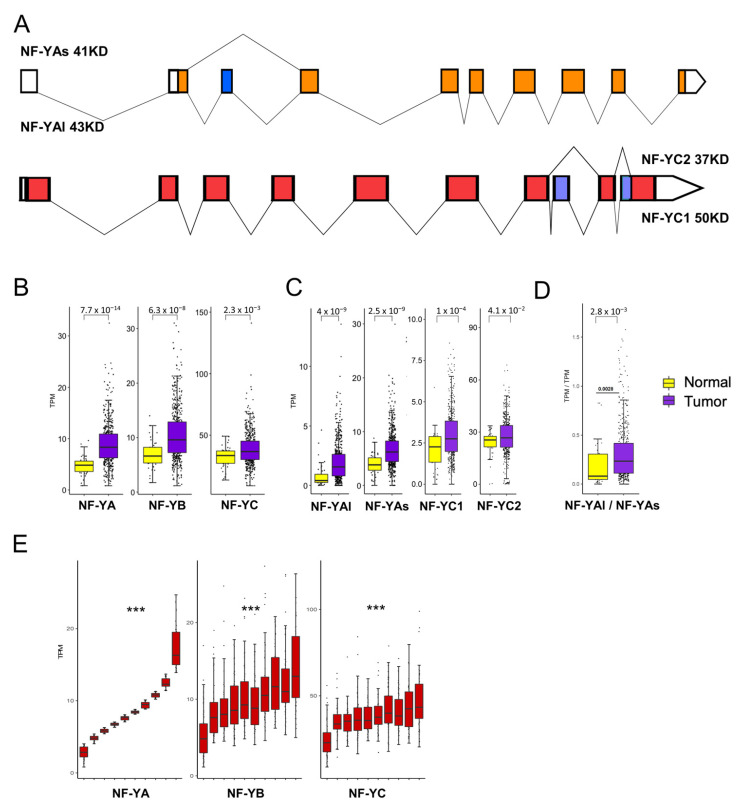
NF-Y subunits are overexpressed in HNSCC. (**A**). Scheme of NF-YA and NF-YC alternative transcripts. (**B**). Box plots of the expression levels of the three NF-Y subunits at gene level in the TCGA HNSCC dataset, measured in TPMs. (**C**). As in A, the NF-YAs, NF-YAl and NF-YC isoform levels were analyzed. NF-YC2 corresponds to the 37 kD isoform; NF-YC1 to the 50 kD isoform. (**D**). Box plots of the expression levels of the ratio of the two NF-YA isoforms. (**E**). Expression of NF-YB/NF-YC (right panels) according to 10 ranked bins of increasing NF-YA levels (left panels). *p* values were calculated using the Wilcoxon rank sum test: *** *p* value < 0.0001.

We analyzed the expression of NF-Y subunits at the gene and isoform levels (Figure 2A). We confirmed that NF-YA is indeed increased in cancer compared with normal samples (*p* value = 10^−14^) (Figure 2B). Interestingly, NF-YB was increased (*p* value = 10^−8^) and NF-YC showed slightly elevated levels (*p* value = 10^−3^). Next, we analyzed the splicing isoforms of NF-YA and NF-YC. Figure 2C shows that the “short” NF-YAs were predominant in normal cells; in tumors, both NF-YA isoforms were substantially increased (*p* value = 10^−9^); as a consequence of this, the NF-YAl/NF-YAs ratio changed slightly in favor of NF-YAl (Figure 2D). As for NF-YC, the expression of the predominant 37 kD NF-YC2 was not changed in tumors; the 50 kD isoform, NF-YC1, increased (*p* value = 10^−4^) but it was still far less expressed than the 37 kD isoform (Figure 2C).

Because of the concomitant increase in the levels of the three subunits, we verified whether there was a correlation of expression among them. To do so, we ranked all tumor samples for the levels of NF-YA mRNA expression, dividing them in 10 bins; we then matched the expression of NF-YB and NF-YC: as shown in Figure 2E, there was a progressive increase of these subunits from NF-YA^low^ to NF-YA^high^ expressing tumors, with a robust statistical significance. We conclude that NF-YA, NF-YB and, to a lesser extent, NF-YC levels were concomitantly increased in HNSCC with respect to normal tissues, and that the major isoforms expressed in tumors were NF-YC 37 kD and NF-YAs, although an increase of NF-YAl was clearly recorded.

### 3.3. Expression of NF-Y Isoforms in HNSCC Cell Lines

**Figure 3:**
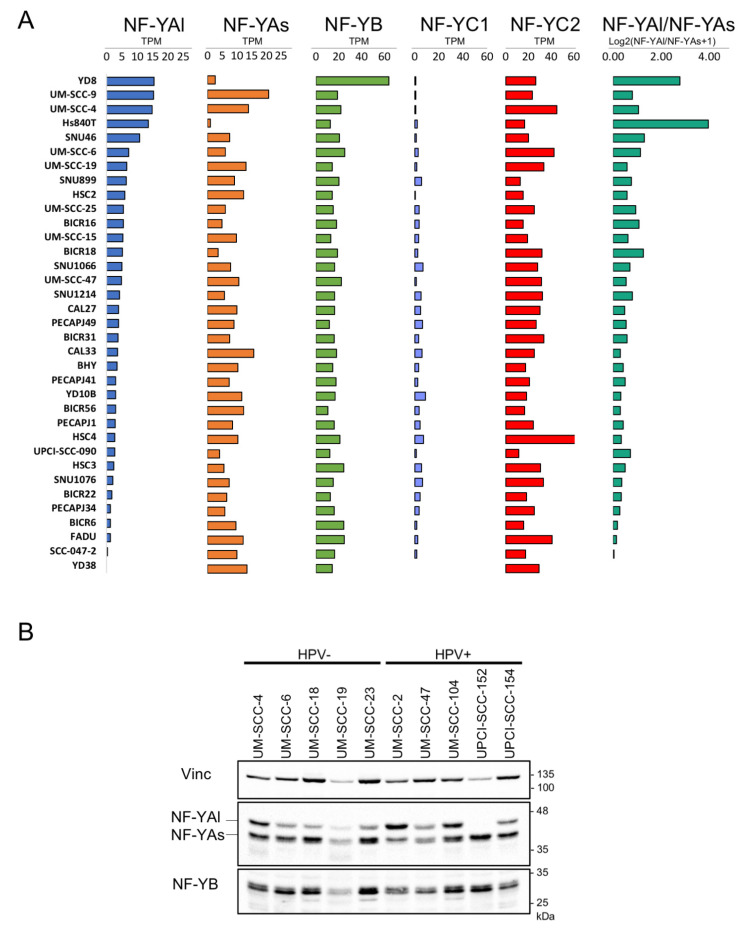
Expression levels of NF-Y mRNA and protein isoforms in HNSCC cell lines. (**A**). Expression levels of NF-Y subunits (expressed in TPMs) and NF-YA isoforms ratio in 35 cell lines of HNSCC. (**B**). Western blot analysis of NF-YA and NF-YB in the indicated HNSCC cell lines.

To correlate the mRNA and protein levels of the isoforms, particularly NF-YAs and NF-YAl, we analyzed their relative levels in RNA-seq data from 35 HNSCC cell lines: the vast majority (26) had a large excess of NF-YAs: only Hs840T, YD8 and SNU46 had higher amounts of NF-YAl (Figure 3A). NF-YB mRNAs were relatively well balanced, with the exception of higher levels in YD8. As for NF-YC, the pattern is relatively uniform, with most lines lacking, or having very low expression of the 50 kD (NF-YC1) isoform.

We performed western blot analysis of the NF-Y subunits in several HNSCC cell lines, both positive and negative for HPV. As shown in Figure 3B, cells showed variable levels of the two NF-YA isoforms, with a prevalence of the short isoform. For UM-SCC-4, UM-SCC-6, UM-SCC-19 and UM-SCC-47, a comparison with the available RNA-seq data (Figure 3A) indicated good concordance: NF-YAs was predominant in UM-SCC-19 and UM-SCC-47, and balanced levels were observed in UM-SCC-4 and UM-SCC-6. Overall, there was no obvious skewing according to the HPV status of the lines. As for NF-YB, we noticed some variation in the protein levels among the lines, somewhat superior to the relatively invariant mRNA levels. Altogether, these data indicate that there is a good agreement between the mRNA and isoforms levels in HSNCC cell lines.

### 3.4. Analysis of NF-Y Subunits Levels According to Somatic Mutations in HNSCC

**Figure 4:**
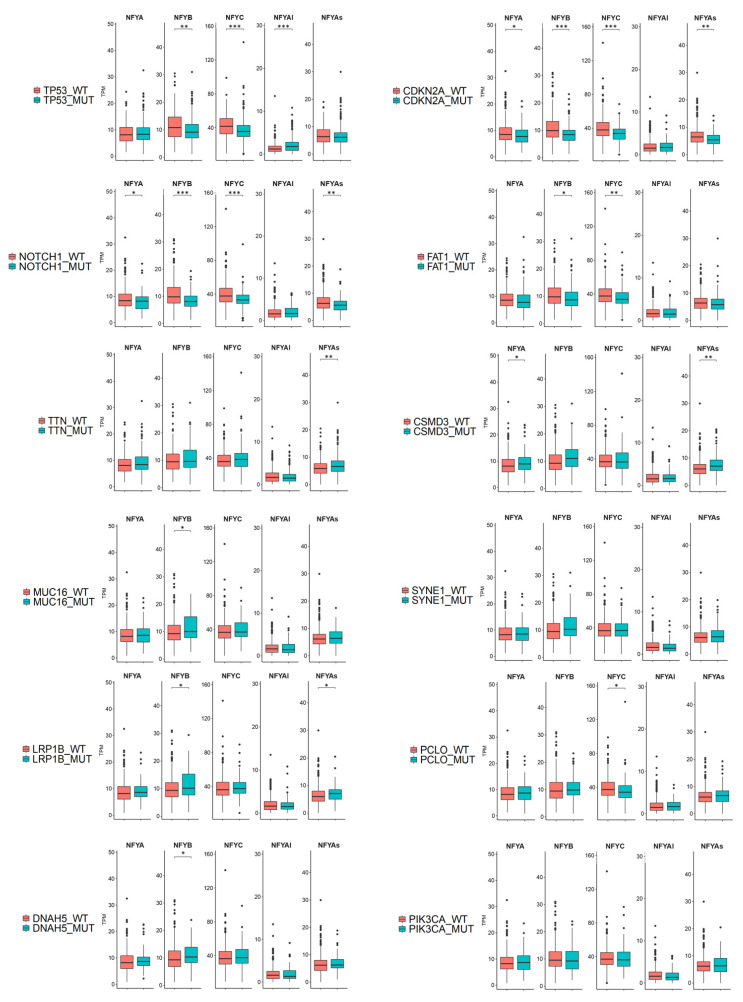
Expression levels of NF-Y isoforms according to common mutations in HNSCC. Relative levels of expression of the NF-Y subunits and isoforms (TPMs) in HNSCC tumors presenting the 12 most common mutations, as indicated in the different Panels. *p* values were calculated using a Wilcoxon signed-rank test. * *p* value < 0.05; ** *p* value < 0.01; *** *p* value < 0.001.

TCGA identified somatic mutations in 506 tumors [6]. Here, we considered only the 12 most frequent mutations present in >80 samples (Figure S1), assessing the NF-Y subunits expression in their relative cohorts (Figure 4). Mutations of CDKN2A (p16) and NOTCH1 were associated with a statistically relevant decrease in NF-YAs, NF-YB and NF-YC; the same was seen in TP53 mutants, but in this case with a substantial increase in NF-YAl. Other changes, less statistically significant, were an increase in NF-YB in CSMD3, an increase in NF-YAs in TTN and CSMD3, and a decrease in NF-YC in PCLO mutants. TCGA has compiled a list of cancer driver genes [37] and the Ciccarelli group has further refined this by pointing out potential false positive drivers [38], based on numerous validation criteria (http://ncg.kcl.ac.uk/false_positives.php, accessed on 10 May 2020). We analyzed the above data and noted that altered expression of NF-Y subunits was most obvious in samples with mutations of *bona fide* drivers (TP53, CDKN2A, NOTCH1, FAT1) but less so in samples with potential “false positive” mutations. We concluded that selected cancer-driving mutations are associated with altered expression, both positive and negative, of NF-Y subunits.

### 3.5. Classification of All TCGA HNSCCs in Four Subtypes

Overexpression of NF-Y subunits could be limited to one or more of the HNSCC subtypes. TCGA has so far reported the partitioning of 279 of the 522 tumors for which RNA-seq data are available [6]. As we have previously done in our analysis of breast, lung and liver cancers, before proceeding with further analysis, we classified all TCGA tumors; to do so, we used the gene signature described by Walter et al. [3]. Table S2 contains our classification of all TCGA tumors. Figure S2A shows the results, depicted as Venn diagrams, of the old and our extended classification: the Atypical and Mesenchymal classes maintain the same proportions, but modest skewing is present in Basal and in Classical, the least frequent subtype. A heatmap of our classification was constructed based on a predictor centroid of 838 genes derived from the Walter classification [3]: this is shown in Figure S2B, confirming a visible clustering of the four major subtypes. In particular, Figure S2C shows the expression levels of typical markers of each subtype [3,6]: Atypical (RPA2, E2F2, FGFR3), Classic (SOX2, NFE2L2, KEAP1, PIC3CA, AKR1C1) and Mesenchymal (PDFGRA, PDGFRB, TWIST1) were partitioned as expected. Of the three basal markers, only TGF-A showed the expected pattern, while the expression of TP63 was higher in classic tumors (Figure S2C). Overall, our extended classification of all TCGA HNSCC tumors was robust and it represented the basis for further analysis.

### 3.6. Expression of NF-Y Isoforms in HNSCC Subtypes

With the complete HNSCC RNA-seq dataset on hand, we performed individual analyses of the RNA-seq of the four subtypes. Venn diagrams of the overlaps are shown in Figure S3A (left panel), and lists of the differentially expressed genes are presented in Table S2. The common set of 257 genes upregulated in all subtypes lacks NF-Y sites (Figure S3A right panel) and retains the features described in the global analysis, notably extracellular matrix terms (Figure S3A bottom panel). As for subtype-specific transcription factor binding sites, distinct matrices were enriched, with *p* values being borderline significant, except for Mesenchymal subtype (Figure S3B). We then analyzed the Gene Ontology terms enriched in individual subtypes (Figure S3C): the signatures of Classical and Atypical subtypes have low statistical significance; Basal and Mesenchymal show the expected terms: extracellular matrix and collagen in the latter, cornified envelope and keratinization in the former (Figure S3C). Overall, while the CCAAT box was enriched in promoters of genes overexpressed in HNSCC at large (Figure 1), it was not in specific subtypes.

**Figure 5:**
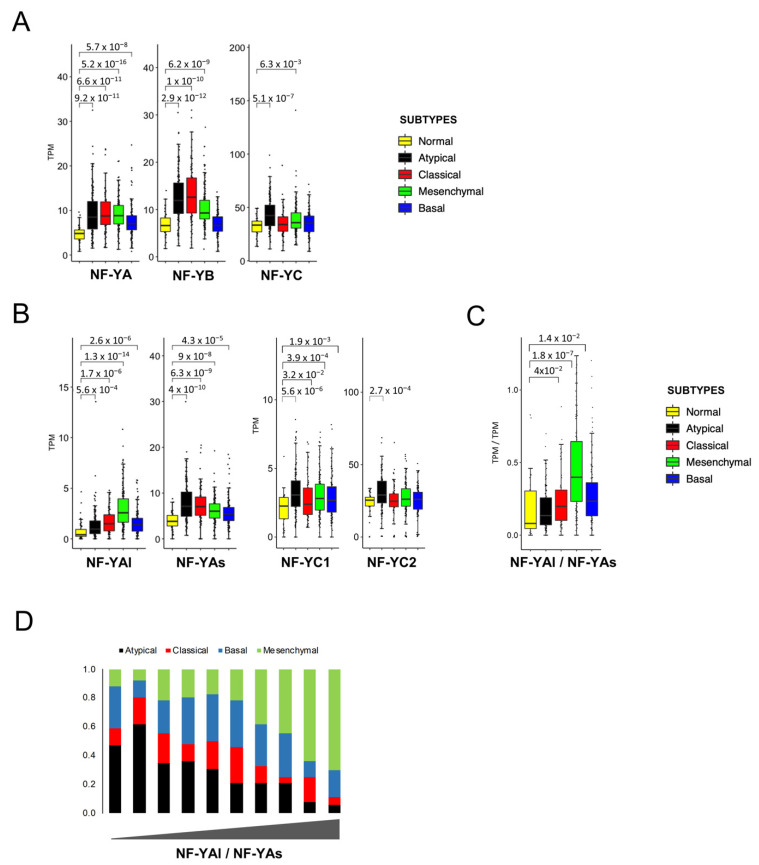
NF-Y subunit expression in HNSCC subtypes. (**A**). Box plots of expression levels of the three NF-Y subunits at gene level in the four subtypes and normal samples of the TCGA HNSCC dataset, measured in TPMs. (**B**). As in A, with the NF-YAs, NF-YAl, NF-YC1 and NF-YC2 subunits. (**C**). Box plots of the expression levels of the ratio of the two NF-YA isoforms. *p* values were calculated using the Wilcoxon rank sum test. (**D**). Partitioning of HNSCC subtypes according to increasing NF-YAl/NF-YAs ratios, divided into 10 bins.

We then investigated the expression levels of the three subunits. Figure 5A shows a global increase in NF-YA in all subtypes, with somewhat variable degrees (*p* values ≤ 10^−8/16^). NF-YB increased at a comparable level in all except Basal tumors; NF-YC was unchanged, except in Atypical tumors, where it is significantly (*p* value = 10^−7^) increased (Figure 5A). The data on NF-YA and NF-YC isoforms are shown in Figure 5B: NF-YAs increased in all, particularly in Atypical tumors (*p* values = 10^−5/10^); interestingly, the most robust increase was scored for NF-YAl in Mesenchymal tumors (*p* value = 10^−14^). As a consequence, the NF-YAl/NF-YAs expression ratio is changed substantially in this subtype (*p* value = 10^−7^) (Figure 5C). As for NF-YC, the increased levels were mostly due to the less expressed NF-YC1 50 kD isoform in Atypical tumors (Figure 5B).

In previous studies on breast and lung tumors, the NF-YAl/NF-YAs ratios were clinically more useful than the levels of the single isoforms. Specifically, partitioning of BRCA cell lines in clusters according to their NF-YAl/NF-YAs ratios, and determining the respective differentially expressed genes, allowed us to derive useful predictor centroids, which were subsequently validated in tumors [24]. This was not possible with HNSCC cell lines, due to the paucity of those with high levels of NF-YAl (Figure 3). We then decided to take a different approach and directly partitioned all samples in 10 bins, according to the ranking of their NF-YAl/NF-YAs ratios. Figure 5D shows that tumors with high ratios (NF-YAl^high^) were mostly (65–69%) of the Mesenchymal subtype, while Atypical tumors were only 4–5%. On the contrary, tumors with low ratios (NF-YAs^high^) were mostly (48–49%) Atypical, with very few Mesenchymal tumors. Basal and Classical tumors had intermediate NF-YAl/NF-YAs ratios. 

In summary, overexpression of NF-YA and NF-YB was generally widespread, with some specificities: NF-YAl/NF-YAs ratios were high in Mesenchymal tumors, due to increased levels of NF-YAl, and low in Atypical tumors, with no increase in NF-YB in Basal and an increase in NF-YC isoforms in Atypical tumors.

### 3.7. Analysis of HPV-Positive Tumors

**Figure 6:**
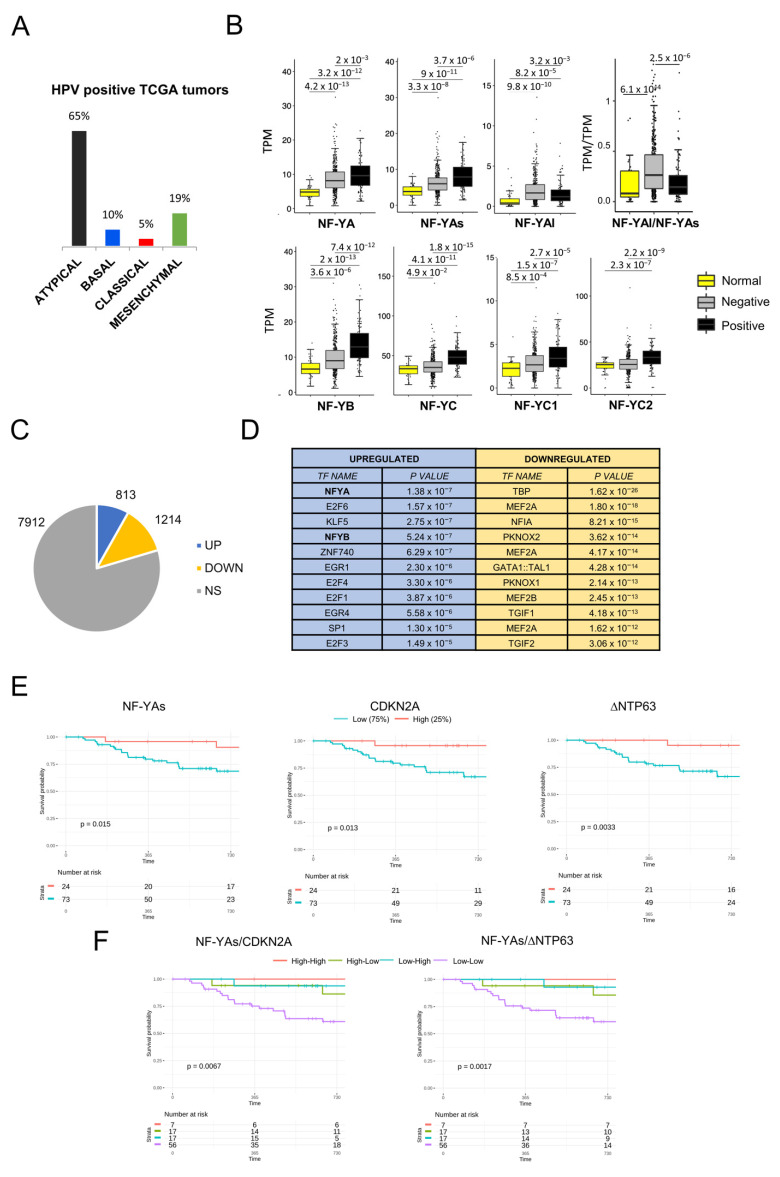
Analysis of NF-Y subunits expression in HPV-positive HNSCC. (**A**). Partitioning of all TCGA HNSCC subtypes according to HPV positivity. (**B**). Box plots of the expression levels of NF-Y subunits at gene and isoform levels in the TCGA HPV-positive and HPV-negative HNSCC, measured in TPMs. *p* values were calculated using the Wilcoxon rank sum test. (**C**). Up- and downregulated genes in HPV-positive versus normal tissues. NS, not significant. (**D**). Pscan analysis of enriched TFBS in promoters (−450/+50 bps from the TSS) of up- and downregulated genes in HPV-positive HNSCC tumors. (**E**). Progression-free interval curves of the survival probability of HNSCC tumors with stratification according to the expression of NF-Y subunits. (**F**). Progression-free interval curves of the survival probability of HNSCC tumors with stratification according to the combination of expression of NF-YAs and CDKN2A or ΔNTP63.

A considerable number of HNSCC, mostly oropharyngeal cancers, are associated with infection with human papilloma virus (HPV) [39,40,41,42], as reviewed in [43,44,45,46]. Firstly, we verified that the 98 HPV-positive tumors present in our new classification were mostly classified as Atypical tumors (65%, Figure 6A), as previously shown by TCGA [6]. We then determined the expression of NF-Y subunits in this cohort. The box plots of global subunits levels in HPV-negative and HPV-positive tumors versus normal samples showed a generalized increase (Figure 6B, left panels). In the central panels, we scored the NF-YA isoforms: NF-YAl was lower, NF-YAs was higher and, as a consequence, the NF-YAl/NF-YAs ratio dropped in HPV-positive tumors (Figure 6B, right panel). In parallel, we identified differentially expressed genes in this cohort, with the same criteria used above. Figure 6C shows that 813 genes were overexpressed and 1214 were downregulated. TFBS analysis by Pscan detected the NF-Y matrix at the top in upregulated genes, with E2F and the sites of KLF5, Znf740, EGR1/4 and Sp1 (Figure 6D, left panels). In downregulated genes, the promoters had other matrices, such as TATA, MEF2 and NFI (Figure 6D, right panels). As for GO pathways, together with the extracellular matrix terms found in the general analysis, we found cell-cycle genes, which was expected based on the concomitant presence of the NF-Y and E2F matrices in the promoters (Figure S4). Therefore, we detected a distinct gene expression profile in HPV-positive tumors, with CCAAT, E2F and motifs of cell-cycle genes; NF-YAs and NF-YB/NF-YC subunits were clearly overexpressed. Note that these features are different from those scored in Atypical tumors at large.

We then considered the clinical data and measured the PFI of HPV-positive tumors. We stratified the data according to the levels of NF-Y subunits (top 25% versus lowest 75%): NF-YA total and NF-YAl were not significant, but NF-YAs correlated with a better prognosis (Figure 6E, left panel). As positive controls, we evaluated CDKN2A, whose high levels are known to be protective [6], as well as ΔNp63, which we recently demonstrated to be increased in HPV-positive by the E6 viral protein [47]; both genes are indeed very significantly protective (Figure 6E, middle and right panels). Finally, pairwise combination of curves of NF-YAs with CDKN2A or ΔNp63 further confirmed this point (Figure 6F). We concluded that overexpression of NF-YAs in HPV-positive tumors correlated with a CCAAT-driven, proliferative signature associated with better patient outcomes.

### 3.8. Deconvolution of Single-Cell RNA-Seq Analysis

All TCGA data were from bulk tumors, which are intrinsically heterogeneous in terms of cellular populations. Hence, it was not possible to determine precisely which population expressed the NF-Y isoforms. Puram et al. reported on an analysis of single-cell RNA-seq from HNSCC [28], providing a breakdown of the TCGA classification and introducing important concepts: (i) the prevalence of fibroblasts in Mesenchymal tumors; (ii) the presence of p-EMT cells, specifically in Basal and Mesenchymal tumors; and (iii) the identification of gene signatures, including those for p-EMT and cancer-associated fibroblasts (CAFs). 

**Figure 7:**
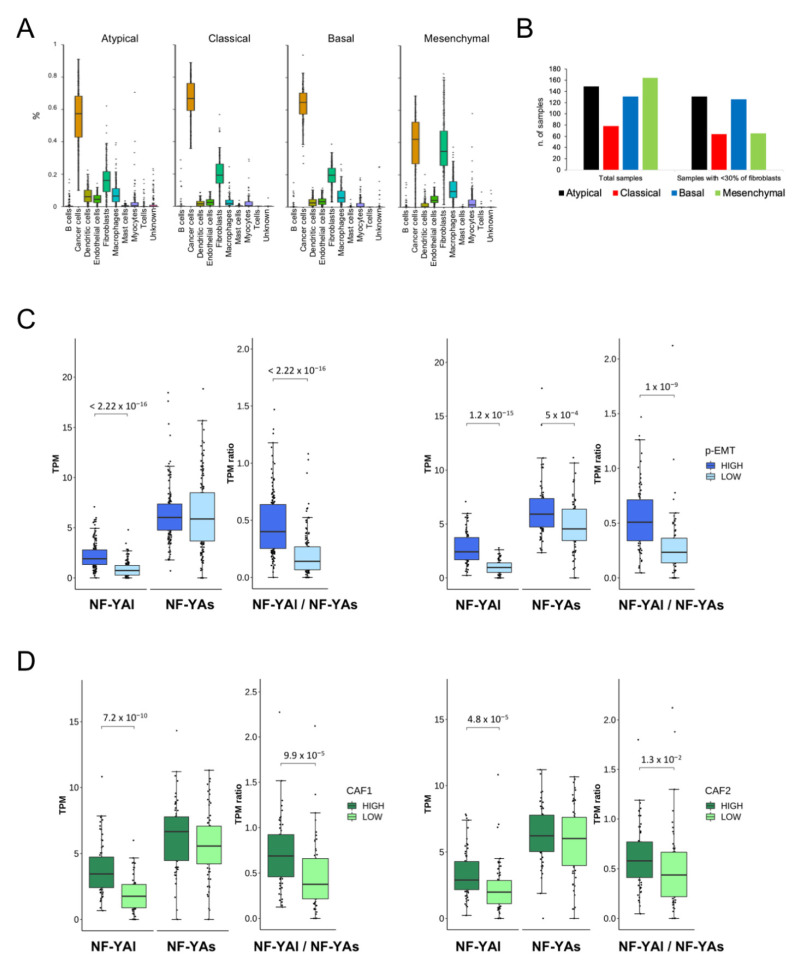
Correlation of NF-YA isoforms according to deconvolution of the scRNA-seq analysis. (**A**). Proportion of the individual cell populations, as predicted based on scRNA-seq deconvolution of tumors, within the TCGA HNSCC subtypes. (**B**). Subtype distribution of all TCGA tumor samples before (left panel) and after (right panel) elimination of samples with a predicted fibroblasts population of >30%. (**C**). Left panels: box plots of NF-YA isoforms and NF-YAl/NF-YAs ratio, according to the p-EMT signature: total TCGA samples are divided into high and low. Right panels: As in C, except that the analysis was limited to Basal and Mesenchymal tumors of the TCGA classification. (**D**). As in C (left panels), except that CAF1 (left panels) and CAF2 (right panels) signatures were used in the total TCGA samples. *p* values were calculated using the Wilcoxon rank sum test.

Fibroblasts are known to mainly express NF-YAl [10]; thus, it was important to assess whether the abundance of NF-YAl found in Mesenchymal tumors (Figure 5B) could be ascribed exclusively to this population of cells. To solve the issue, we deconvoluted the single-cell RNA-seq data and partitioned the subtypes according to the cellular components (cancer cells, fibroblasts, myocytes, macrophages etch). The results are shown in Figure 7A: cancer cells, which are prevalent, were equally distributed in all subtypes, whereas fibroblasts were specifically abundant in the Mesenchymal subtype, as expected from Puram’s analysis. We then analyzed all TCGA samples, after elimination of those particularly abundant in fibroblasts (>30% of cellularity) according to the deconvolution: Figure 7B shows that indeed most, but not all, of the evicted samples belong to the Mesenchymal subtype. We partitioned the remaining samples according to their p-EMT signature (High and Low), as described by Puram et al. [28]: the box plots of Figure 7C (left panels) show that a high NF-YAl and NF-YAl/NF-YAs ratio, but not NF-YAs, correlated with the signature. In their analysis of TCGA data, Puram et al. focused on the Basal and Mesenchymal subtypes; we thus repeated the analysis focusing on these two subtypes, excluding Classical and Atypical tumors, and indeed found a correlation very similar to the global analysis (Figure 7C, right panels). Therefore, NF-YAl^high^ and a high NF-YAl/NF-YAs ratio were associated with cancer-promoting cells with a partial mesenchymal phenotype. 

Finally, we analyzed NF-YA isoforms levels in cancer-associated fibroblasts of the Mesenchymal subtype, as defined by Puram’ CAF1 and CAF2 specific gene signatures. We observed a correlation with high NF-YAl levels, as well as the NF-YAl/NF-YAs ratio, for both signatures (Figure 7D). In conclusion, we showed that high NF-YAl, but not NF-YAs, was associated both with p-EMT cells and CAFs within tumors in vivo.

## 4. Discussion

The increased expression of NF-YA in epithelial tumors is becoming obvious, as other Authors have shown [19,20,21,22,23], and as we have progressively been reporting in our systematic TCGA-based analysis [24,25,26,27]. The results shown here further confirm the abundance of NF-Y sites (CCAAT) in the promoters of genes overexpressed in cancer, the increased expression of the “short” NF-YA in epithelial tumors and of “long” NF-YA in tumors expressing mesenchymal markers. In addition, we found (i) global overexpression of the NF-YB/NF-YC subunits, with clinical implications, and (ii) the association of selected somatic mutations and HPV infection with altered expression of the subunits. Most importantly, deconvolution of single-cell RNA-seq led us, for the first time, to associate NF-YAl expression with the p-EMT subpopulation.

CCAAT boxes have been routinely found in promoters of genes overexpressed in cancer, first in microarray profiling experiments [8] and subsequently in RNA-seq data. CCAAT genes are associated with pro-proliferative GO terms, either globally (breast) or in subpopulations, such as in the lung and liver [24,25,26,27]. We found them here in HPV-positive tumors, in agreement with NF-Y being essential for cell-cycle and metabolic genes associated with tumorigenesis [18]: CCAAT was found with E2Fs sites, in line with their role and overexpression in cancers. In HNSCC at large, instead, we found extracellular matrix/collagen and general mesenchymal terms in upregulated genes. E2Fs motifs were not enriched. This might reflect the abundance of fibroblasts, primarily, but not exclusively, in Mesenchymal tumors. As expected, CCAAT was not found in the promoters of genes downregulated in HNSCC: despite their frequency in promoters, and their recent inclusion in the DNA elements that govern transcriptional start site selection [48], this confirmed that promoter configurations of “cancer” genes—with CCAAT—are structurally different from downregulated genes, which are often CCAAT-less.

A notable difference with respect to our analysis in breast and lung cancers is that NF-YB and, to a lesser extent, NF-YC are overexpressed in tumors. The number of NF-YB mRNA molecules appears to approach that of NF-YC, the most expressed of the subunits. This has a precedent in liver cancers [27] and in one study on NF-YC in gliomas [49]. Overexpression of NF-YB could impact cancer cells, as this subunit was shown to have a pro-survival effect, in partnership with E2F1 [50]. Regulation of the localization of HFD subunits might also be relevant. No specific data are available on HNSCC lines, but NF-YB is intrinsically nuclear in other epithelial cells, while NF-YC is also cytoplasmatic, being transferred to the nucleus through NF-YB dimerization [51,52]. Thus, higher NF-YB levels could increase heterodimer formation in the nucleus, which is, in turn, met by higher levels of NF-YA to form functional CCAAT-binding trimers. Note that a direct relationship between increased levels of NF-YA mRNA and protein levels has been documented in gastric cancer [23] and we have shown that this was also observed in HNSCC cell lines. Furthermore, expression of the NF-YC protein is affected by the levels of the oncogenic lncRNA SNHG3 in SCC-9 cells, via decoying of the RNA-binding protein ELAV1L [53].

NF-Y subunits are rarely mutated in tumor samples. We evaluated the association of NF-Y levels with mutations frequently detected in HNSCC and noted the link with p53. NF-YAs and HFD subunits are comparatively less expressed in p53, NOTCH1 and CDKN2A (p16) mutated tumors. Furthermore, we reported an increase in NF-YAl. As for p53, 60–65% of HNSCC are mutated [4,5,6,54,55,56], whereas squamous cell carcinomas of the skin and lung reach 85–90% [57]. Infection by the HPV virus is a powerful driving force in the pathogenesis of HNSCC, particularly those of the oropharynx, with approximately 70% being HPV16-positive [38,39,40,41,45]. A growing body of genetic and clinical evidence indicates that HPV-positive tumors are different from HPV-negative tumors. They carry wtp53, while the vast majority (>80%) of HPV-negative tumors are mutated in p53 [6]. HPV produces viral proteins (E6, E7) which inactivate the function of wtp53 by several mechanisms [43,44,45]. In essence, p53 in HNSCC is either mutated or inactivated by viral proteins. Both mechanisms were shown to impact on the NF-Y regulome: p53 mutations, specifically those with gain-of-function, directly activate several NF-Y targets [58,59,60,61,62,63]. As for viral proteins, NF-Y activates the E6 promoter of HPV18 in HeLa cells, via a proximal promoter CCAAT box [64]. It remains to be seen whether an increased level of NF-Y induces HPV16 E6/E7 expression. In HPV-positive tumors, expression of all subunits was substantially increased, and positive feedback could increase the oncogenic potential of E6/E7 proteins. Inactivation of E6 reactivates wtp53 protein, causing either direct apoptosis or sensitizing cells to anti-cancer drugs [65,66,67]. Another player is the related p63, specifically the ΔNp63 isoform, which is predominant in squamous cell carcinomas; p63 is a master gene of stratified epithelia [68], whose role in HPV-positive tumors is not entirely clear. We recently showed that E6 activates the expression of ΔNp63 in HPV-positive cells [46], and found a correlation here with NF-YB/NF-YC//NF-YAs. The promoter of ΔNp63 contains two CCAAT elements and is regulated by NF-Y [69]: we are thus tempted to speculate that E6 and the increased NF-Y levels lead to overexpression of ΔNp63. In turn, increased expression of p63 would maintain epithelial identity, preventing epithelial to mesenchymal transition: this would be in line with the clinical data of Figure 6, showing the worst prognosis in patients whose ΔNp63 and NF-YAs levels are low. At the opposite end, high levels of mutp53, as found in most HPV-negative tumors, could subvert the expression of the epithelial markers normally controlled by ΔNp63, with loss of epithelial identity. A further issue is the co-regulation of the three subunits. In HPV18-positive HeLa cells, removal of NF-YA by RNAi leads to increased HFD levels, and, vice versa, NF-YB inactivation entails upregulation of NF-YA [64]. In HPV-positive tumors, all three subunits are upregulated: thus, the feedback loop is apparently interrupted. The role of viral proteins on subunit levels, mutp53 and ΔNp63 awaits further biological confirmation.

The two major NF-YA splicing isoforms differ in the Gln-rich *trans*-activation domain (TAD), with NF-YAl harboring 28/29 extra amino acids coded by Exon 3: both isoforms retain subunit interaction and DNA-binding domains, leading to the indistinguishable CCAAT-binding of the isoforms. They have different activation potential [10,70]. Short NF-YA is the most abundant, both in normal tissue and in HNSCC. This isoform is also predominant in all epithelial cancers we have examined so far: we are starting to believe that this is linked to the epithelial identity of the cells. A subset of Claudin^low^ breast tumors in which NF-YAl predominates corresponds to the most aggressive, EMT-prone subset. Indeed, it was recently proposed that the Claudin^low^ group represents a fifth subtype in BRCA [71]. We found a similar situation in the HNSCC Mesenchymal subtype, with a correlation between EMT markers and NF-YAl^high^ and high NF-YAl/NF-YAs ratios.

TCGA provides data from bulk tumors. The seminal study of Puram et al. called for a simplification of the current classification, indicating that Basal and Mesenchymal subtypes could be classified into a single subtype, with Mesenchymal being a subclass with an abundant component of stromal cells [28]. Most importantly, the study showed evidence of the existence of p-EMT cells, located at the leading edge of tumors. They also described gene signatures for CAFs. By deconvoluting single-cell RNA-seq data with the use of the p-EMT and CAF signatures, we established a correlation between NF-YAl, but not NF-YAs, and the typical markers of CAFs, which are important components of the tumor microenvironment, including in squamous cell carcinomas [72]. We also provide the first indication that p-EMT, a metastasis-prone population, correlates with NF-YAl, and their NF-YAl/NF-YAs ratios are different from other subtypes (Atypical, Classic). This confirms and extends Puram’s study, which called for the modification of the current classification of HNSCC based on four molecular subtypes. Our results also have potential diagnostic value: on one hand, we have reinforced the necessity of deriving tools to carefully assess the p-EMT signature in primary tumors, which is apparently more revealing than other features based on the current molecular classification; on the other, quantification of the single NF-YA isoforms by RT-PCR might be valuable in the assessment, respectively, of HPV-positive (NF-YAs^high^/protective) and HPV-negative (NF-YAl^high^/deleterious) tumors.

## 5. Conclusions

We have pointed to the role of NF-YAs/HFD in the prognosis of wtp53 HPV-positive tumors and of NF-YAl in p-EMT (Basal/Mesenchymal) cells. Mechanistically, as most EMT genes are CCAAT-less, NF-YAl’s role might be indirect, via activation of EMT-inducing TF(s). We should extend our findings to the p-EMT/NF-YAl connection with other tumors through single-cell RNA-seq data (BRCA, in primis), as well as identifying connections with EMT TF(s). Impairment of NF-Y activity could be desirable in HPV-negative tumors, and attempts in this direction have recently started [73,74].

## Data Availability

The data presented in this study are available on request from the corresponding author.

## Supplementary Materials

The following are available online at https://www.mdpi.com/article/10.3390/cancers13123019/s1, Figure S1. Tumor mutations in HNSCC TCGA data. Available genetic data on 316 TCGA HNSCC were portioned according to the individual mutations, as shown in the table. Figure S2. A. Venn diagram of the TCGA HNSCC dataset with the four subtypes previously categorized [3]. Each circle shows the number with the previous partial partitioning and the new classification presented here. B. Heatmap with clustering of HNSCC samples in four subtypes, according to the 838 gene centroids, kindly provided by V. Walter [3]. C. Box plots of relative mRNA levels of 14 marker genes typical of the four subtypes, as indicated by the color: black (Atypical), green (Mesenchymal), red (Classical), blue (Basal). Figure S3. Analysis of HNSCC subtype-specific gene expression. A. Venn diagrams of upregulated genes in the different subtypes of HNSCC. Right panel: Pscan analysis of enriched TFBS in promoters of the common cohort of upregulated genes. Lower panel: Reactome analysis of the upregulated genes. B. Pscan analysis of enriched TFBS in promoters of the subtype-specific upregulated genes, as indicated by the Venn diagrams. C. Reactome pathways enriched in upregulated genes in the four subtypes of HNSCC, listed according to their *p* value. The list was obtained using KOBAS. Figure S4. Pathways enriched in HPV tumors. Reactome pathways enriched in HPV-positive upregulated genes (upper panel) and downregulated genes (lower panel) listed according to their *p* value. The list was obtained using KOBAS. Figure S5: The uncropped western blotting figure. Table S1. Differentially expressed genes. Table S2. Classification of TCGA samples.
