# Supplementary material for: NF-Y Subunits Overexpression in HNSCC
Source: Cancers (Basel). 2021 Jun 16;13(12):3019. doi: 10.3390/cancers13123019 (PMC8234210; doi:10.3390/cancers13123019)
Supplement: Supplementary file 1 [file cancers-13-03019-s001.zip › cancers-1230951-supplementary/cancers-1230951-Figure S5-original western blotting figure.pdf]

Uncropped western-blot membranes

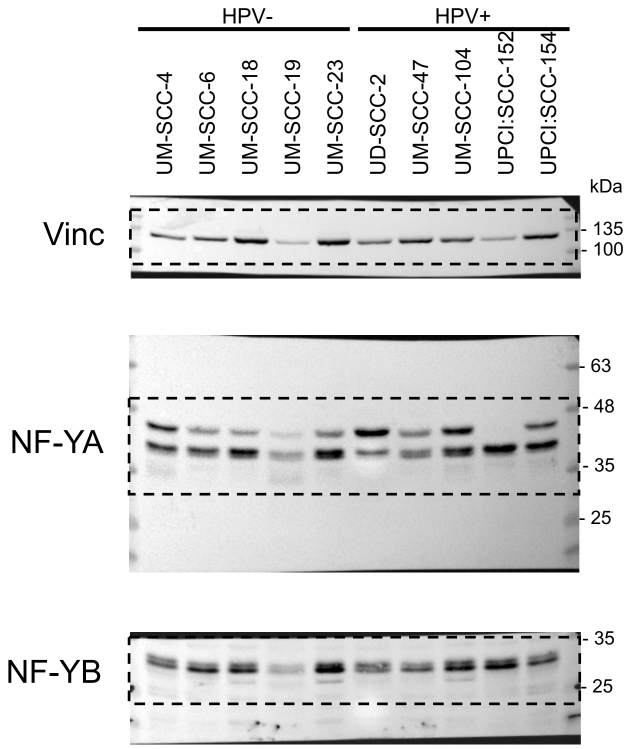

Uncropped western blot membranes referred to Fig 3. Each image represent the merge of the chemiluminescent signal and the colorimetric acquisition of the membrane.
