# Supplementary material for: NF-Y Subunits Overexpression in HNSCC
Source: Cancers (Basel). 2021 Jun 16;13(12):3019. doi: 10.3390/cancers13123019 (PMC8234210; doi:10.3390/cancers13123019)
Supplement: Supplementary file 1 [file cancers-13-03019-s001.zip › cancers-1230951-supplementary/cancers-1230951-Supplementary Figures.pdf]

NF-Y subunits are overexpressed in HNSCC.

Eugenia Bezzecchi[1], Andrea Bernardini[1], Mirko Ronzio[1], Claudia Miccolo[2], Susanna Chiocca[2], Diletta Dolfini [1] and Roberto Mantovani[1]*.

Dipartimento di Bioscienze, Università degli Studi di Milano, Via Celoria 26, 20133 Milano, Italy.
Department of Experimental Oncology, IEO, European Institute of Oncology IRCCS, Via Adamello 16, 20139 Milan, Italy.

* To whom correspondence should be addressed.

Tel. 39-02-50315005

E-mail: mantor@unimi.it

gene	MUT samples	PERCENTAGE
TP53	361	71%
TTN	246	49%
FAT1	122	24%
MUC16	115	23%
CSMD3	113	22%
CDKN2A	107	21%
LRP1B	101	20%
SYNE1	99	20%
NOTCH1	94	19%
PCLO	94	19%
PIK3CA	93	18%
DNAH5	82	16%

Total number of samples = 506

Bezzecchi et al. Figure S1

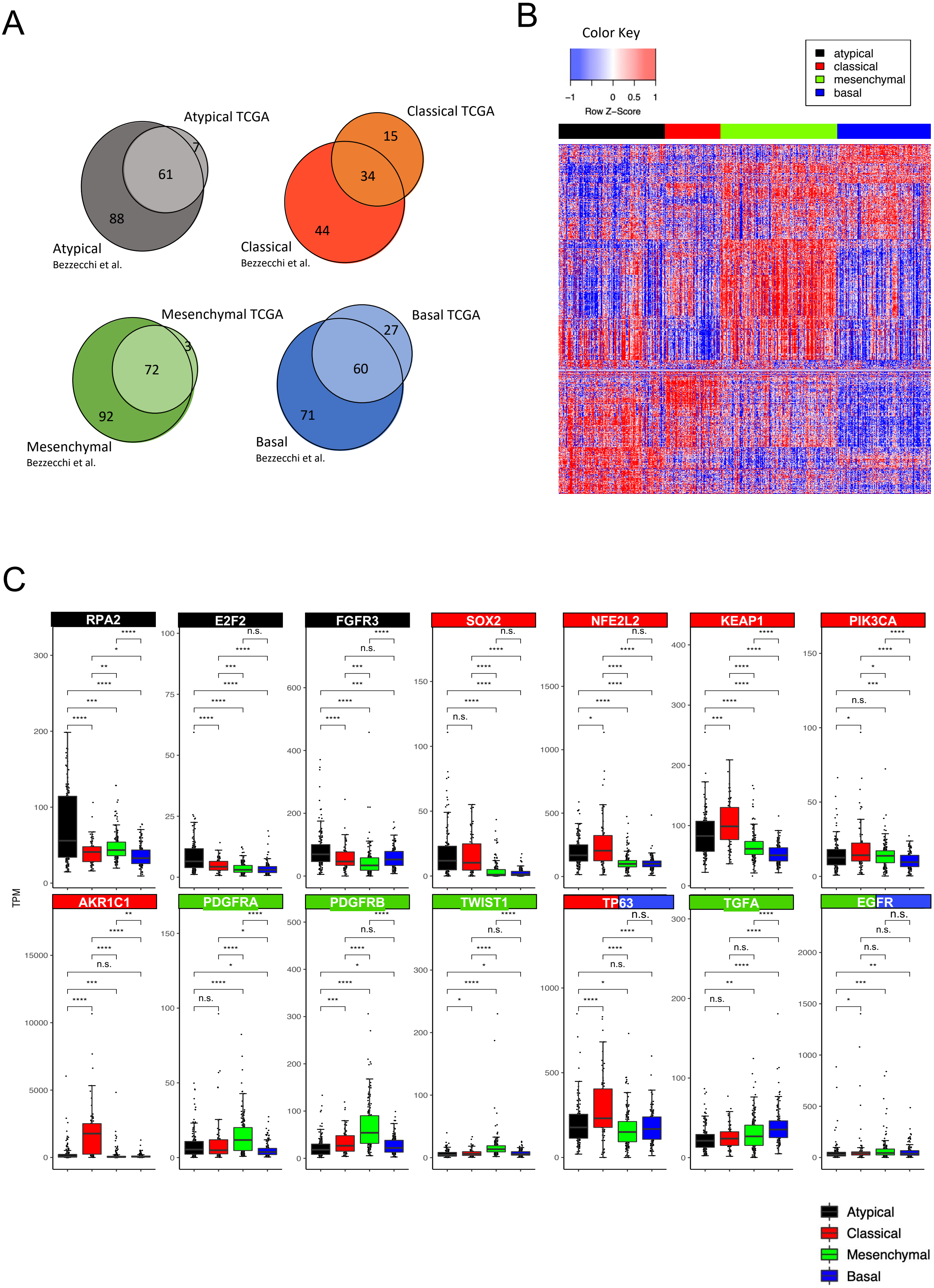

Bezzecchi et al. Figure S2

A

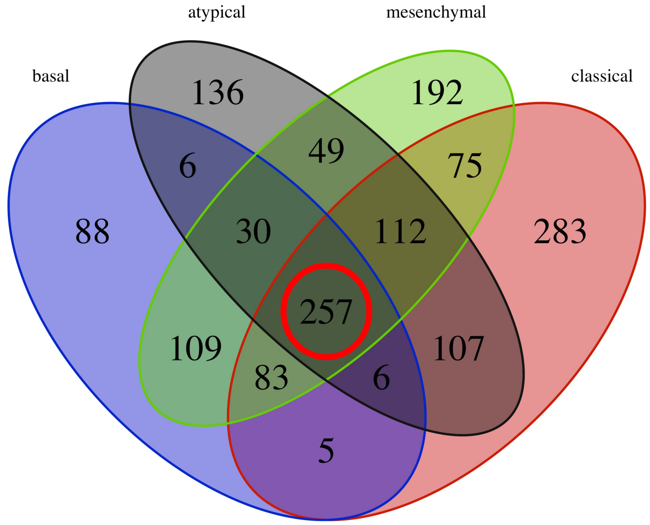

TF_NAME	P_VALUE
RREB1	6.42×10^{-6}
ZNF740	3.91×10^{-5}
USF1	4.42×10^{-5}
TFEB	9.59×10^{-5}
TFE3	1.75×10^{-4}
USF2	2.49×10^{-4}
MLXIPL	3.20×10^{-4}
SREBF2(var.2)	3.53×10^{-4}
Srebf1(var.2)	4.18×10^{-4}
MLX	4.36×10^{-4}

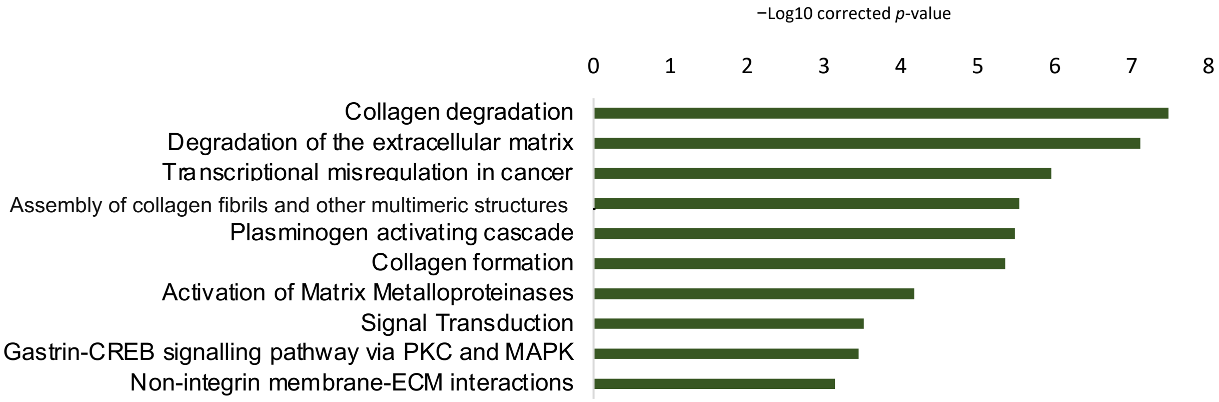

Bezzecchi et al. Figure S3A

B

TF_NAME	P_VALUE
TCF4	5.33×10^{-5}
TCF3	8.74×10^{-5}
ID4	1.06×10^{-4}
PLAG1	4.19×10^{-4}
FIGLA	4.63×10^{-4}
SNAI2	1.09×10^{-3}
ZEB1	1.09×10^{-3}
ZNF263	1.45×10^{-3}
NFKB1	1.46×10^{-3}
TFAP2A	1.66×10^{-3}

TF_NAME	P_VALUE
ZNF263	9.34×10^{-8}
MZF1(var.2)	3.60×10^{-6}
INSM1	5.41×10^{-6}
E2F6	2.17×10^{-5}
EWSR1-FLI1	3.92×10^{-5}
NFKB2	1.63×10^{-4}
E2F3	2.14×10^{-4}
SP1	2.24×10^{-4}
TFAP2A(var.2)	2.33×10^{-4}
E2F4	4.5×10^{-4}

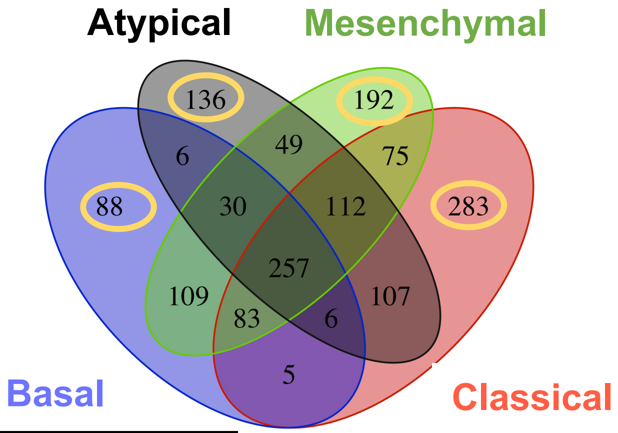

TF_NAME	P_VALUE
JUN::FOS	5.55×10^{-4}
STAT1::STAT2	9.26×10^{-4}
TBP	1.78×10^{-3}
RARA(var.2)	2.02×10^{-3}
TBP	2.12×10^{-3}
RARA::RXRA	3.27×10^{-3}
BATF::JUN	3.58×10^{-3}
STAT1	5.03×10^{-3}
NFKB1	5.20×10^{-3}
MAX	5.66×10^{-3}

TF_NAME	P_VALUE
Gata1	8.68×10^{-4}
ALX3	2.72×10^{-3}
GATA1::TAL1	3.30×10^{-3}
Gata1	3.77×10^{-3}
GATA2	4.12×10^{-3}
SCRT1	4.35×10^{-3}
Gata4	6.41×10^{-3}
HNF1B	6.60×10^{-3}
MIXL1	7.78×10^{-3}
RAX2	8.95×10^{-3}

Bezzecchi et al. Figure S3B

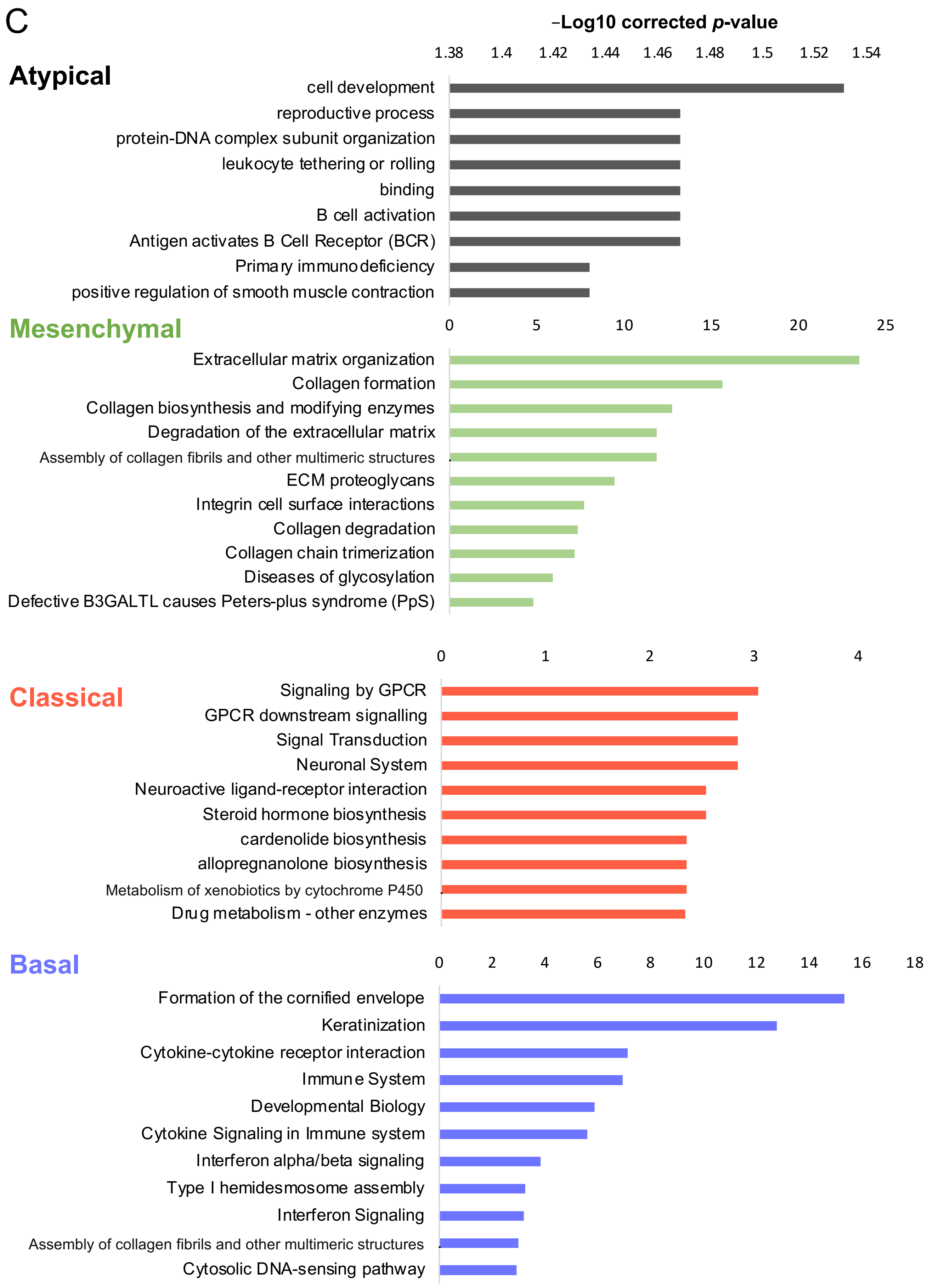

Bezzecchi et al. Figure S3C

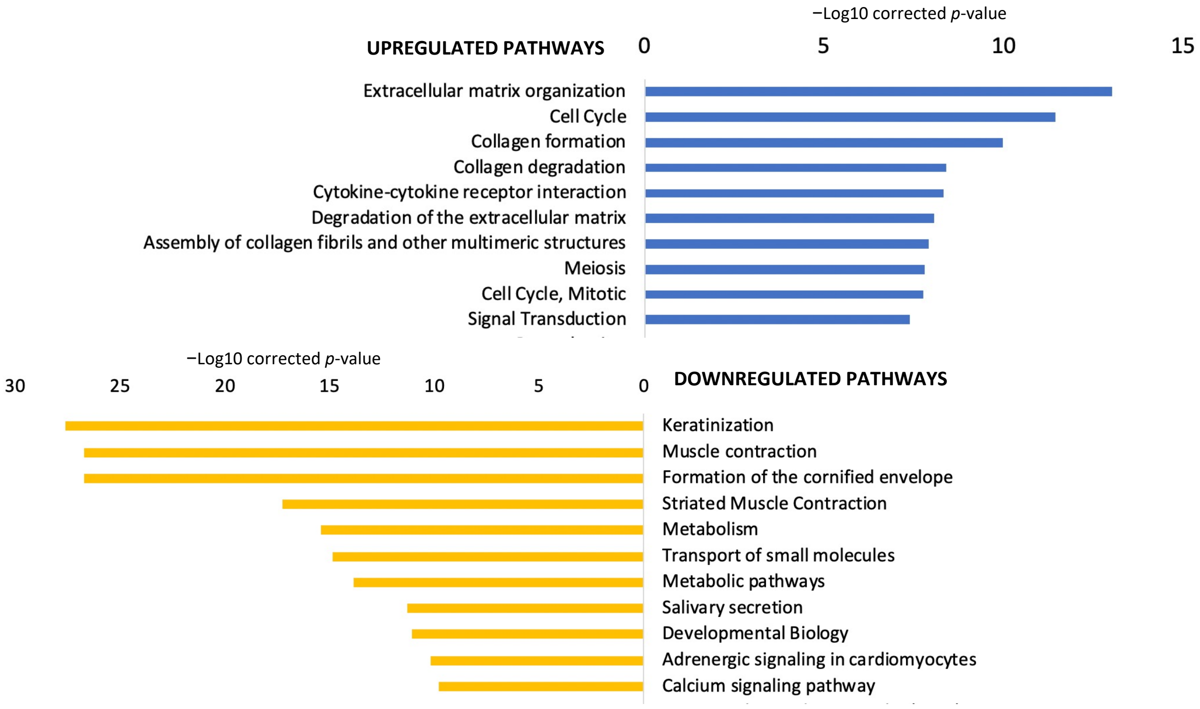

Bezzecchi et al. Figure S4

Figure S1. Tumor mutations in HNSCC TCGA data.
Available genetic data on 316 TCGA HNSCC were portioned according the individual mutations, as shown in the Table.

Figure S2. Venn diagram of the TCGA HNSCC dataset with the four subtypes previously categorized [3]. In each circle, the number with the previous partial partitioning, and the new classification presented here. **B**. Heatmap with clustering of HNSCC samples in four subtypes, according to the 838 genes centroid, kindly provided by V. Walter [3]. **C**. Box plots of relative mRNA levels of 14 marker genes typical of the four subtypes, as indicated by the color: black (Atypical), green (Mesenchymal), red (Classical), Blue (Basal). *p* value: n.s.>0.05, *<0.05, **<0.01, ***<0.001,****<.00001.

Figure S3. Analysis of HNSCC subtype-specific gene expression.
A. Venn diagrams of Up-regulated genes in the different subtypes of HNSCC. In the right Panel, Pscan analysis of enriched TFBS in promoters of the common cohort of up-regulated genes. In the lower Panel, Reactome analysis of the up-regulated genes. **B**. Pscan analysis of enriched TFBS in promoters of the subtypes-specific up-regulated genes, as indicated by the Venn diagrams. **C**. Reactome pathways enriched in up-regulated genes in the four subtypes of HNSCC, listed according to their p-value. The list is obtained using KOBAS.

Figure S4. Pathways enriched in HPV tumors.
Reactome pathways enriched in HPV-positive upregulated genes (Upper Panel) and down-regulated genes (Lower Panel) listed according to their *p* value. The list is obtained using KOBAS.
